# pH-Controlled *Bacillus thuringiensis* Cry1Ac Protoxin Loading and Release from Polyelectrolyte Microcapsules

**DOI:** 10.1371/journal.pone.0045233

**Published:** 2012-09-14

**Authors:** Wenhui Yang, Kanglai He, Jie Zhang, Shuyuan Guo

**Affiliations:** 1 School of Life Science, Beijing Institute of Technology, Beijing, China; 2 State Key Laboratory for Biology of Plant Diseases and Insect Pests, Institute of Plant Protection, Chinese Academy of Agricultural Sciences, Beijing, China; Universidad Nacional Autonoma de Mexico, Instituto de Biotecnologia, Mexico

## Abstract

Crystal proteins synthesized by *Bacillus thuringiensis* (Bt) have been used as biopesticides because of their toxicity to the insect larval hosts. To protect the proteins from environmental stress to extend their activity, we have developed a new microcapsule formulation. Poly (acrylic acid) (PAH) and poly (styrene sulfonate) (PSS) were fabricated through layer-by-layer self-assembly based on a CaCO_3_ core. Cry1Ac protoxins were loaded into microcapsules through layer-by-layer self-assembly at low pH, and the encapsulated product was stored in water at 4°C. Scanning electron microscopy (SEM) was used to observe the morphology of the capsules. To confirm the successful encapsulation, the loading results were observed with a confocal laser scattering microscope (CLSM), using fluorescein-labeled Cry1Ac protoxin (FITC-Cry1Ac). The protoxins were released from the capsule under the alkaline condition corresponding to the midgut of certain insects, a condition which seldom exists elsewhere in the environment. The following bioassay experiment demonstrated that the microcapsules with Cry1Ac protoxins displayed approximately equivalent insecticidal activity to the Asian corn borer compared with free Cry1Ac protoxins, and empty capsules proved to have no effect on insects. Further result also indicated that the formulation could keep stable under the condition of heat and desiccation. These results suggest that this formulation provides a promising methodology that protects protoxins from the environment and releases them specifically in the target insects’ midgut, which has shown potential as biopesticide in the field.

## Introduction

*Bacillus thuringiensis* (Bt) is a Gram-positive, spore-forming bacteria that produces insecticidal crystal proteins named Cry toxins during sporulation. The crystals are synthesized spontaneously as inactive protoxins and are activated by gut protease at high pH in the larval midgut of insect. The activated toxins are considered to be relatively resistant to further protease degradation [1]. Cry toxins are effective in killing a highly specific spectrum of insect species and are widely used as substitutes for broad spectrum chemical insecticides because they are more environmentally friendly [2].

Despite the successful application of Bt for pest control, which presently is approximate 2% of the total insecticidal market [3], the short persistence of *B. thuringiensis* agents after application has become an important influencing factor for its further development. Variable environmental stress, such as UV radiation, rain, and temperature, leads to inactivation or rain-washed of the crystal proteins [4], [5]. Several methods have been investigated to improve the activity and efficiency of these toxins. Cry toxin activity can be potentiated by additional proteins, such as endochitinase [6] and serine protease inhibitors [7]. Modifications in the Cry toxin gene, such as site mutations [8], and hybrid toxins [9], [10] are also effective. In addition, formulation is vital for the success of Bt-based biopesticides. Microencapsulation, a type of formulation, is a potent method conferring resistance to environment stress [11]. In recent years, microcapsules have an increased interest as potential drug carriers, especially for protein drugs. During such therapeutic approaches, protein drugs are directly delivered to the target site and isolated from enzymatic digestion [12]. Furthermore a new kind of viral-assembled monolayer has many potential technological applications in the field of chemical and biological sensors, power devices and catalytic reactive membranes [13]. Polyelectrolyte microcapsules, having controllable stability and high permeability for polar molecules, can be prepared by the layer-by-layer (LbL) self-assembly technique. Polyelectrolyte multilayers can be immobilized on different templates through repeated deposition, mainly due to electrostatic attraction [14], [15]. Hollow capsules are obtained after removal of the core templates. The multilayer shell, designed to specific thicknesses and containing specific components, can be prepared by controlling the species of electrolyte and the layer number. A variety of polymers, such as chitosan, alginate, poly (styrene sulfonate) (PSS), poly (allylamine hydrochloride) (PAH), (polyacrylic acid) polypeptides and polysaccharides, are used as capsule shell components [16]. The compositions of the capsule shells and the core templates have a dominant impact on the properties of the microcapsules. Capsules produced by different method and ingredients demonstrated different activities for improving *B. thuringiensis* residual activity [17]. The hollow microcapsules can be loaded with macromolecules by adjusting the pH value [18] or the salt concentration [19], and the release can be controlled by different factors, such as light, magnetism, pH, salt, glucose and redox [16]. Consequently, the pH is a viable method for controlling the loading and release process.

In this study, we proposed to develop microcapsules by the LbL self-assembly technique with controllable loading and release capabilities for use with Cry1Ac protoxins. CaCO_3_ microparticles were prepared in the presence of PSS, a negatively charged polyelectrolyte. PAH and PSS were chosen as the polyelectrolyte multilayers for their appropriate isoelectric point to achieve pH control. An extensive release of protoxins was observed for the first time at high pH, which corresponds to the pH environment of the larval midgut. The insecticidal activity of the encapsulated Cry1Ac protoxin was detected. In addition, the resistance of protoxins and capsules to the environment factors, such as heat and desiccation was also detected.

## Result

**Figure 1:**
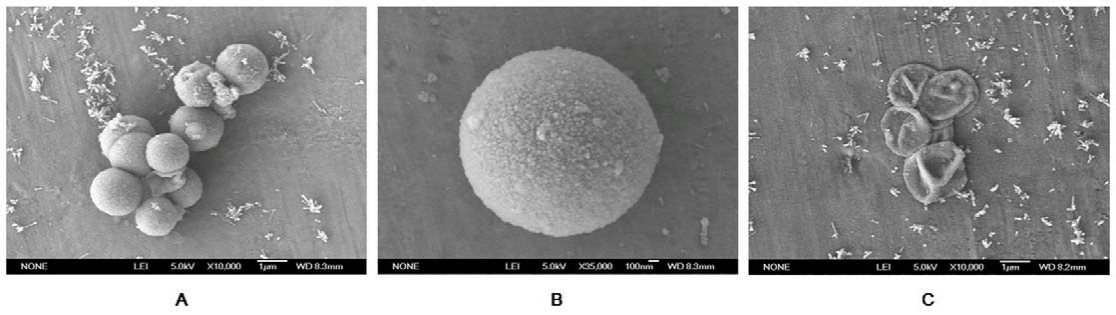
SEM photos of the microcapsules. A and B are capsules with a CaCO_3_ core. C shows capsules after dissociation of the CaCO_3_ particles.

Microparticles with the core of CaCO_3_ were prepared through the LbL methods described above. The SEM micrograph of these microparticles is shown in Fig. 1 (a, b). The microparticles were spherical in shape with a diameter ranging from 2 to 4 µm. After dissociation of the CaCO_3_ particles in 0.2 M EDTA, hollow microcapsules were obtained (Fig. 1c). After removal of the CaCO_3_ core, the size of the obtained hollow microcapsules was slightly larger, compared with the microparticles due to structural collapse. The collapse and creases are typical of hollow capsules, owing to the dissolution of the core and the low mechanical strength of the capsule wall. As an additive, PSS has been shown to influence the crystallization of CaCO_3_ [20], [21]. The size of the microcapsules was appropriate for the insect diet. The microcapsules were stored at 4°C for further use.

**Figure 2:**
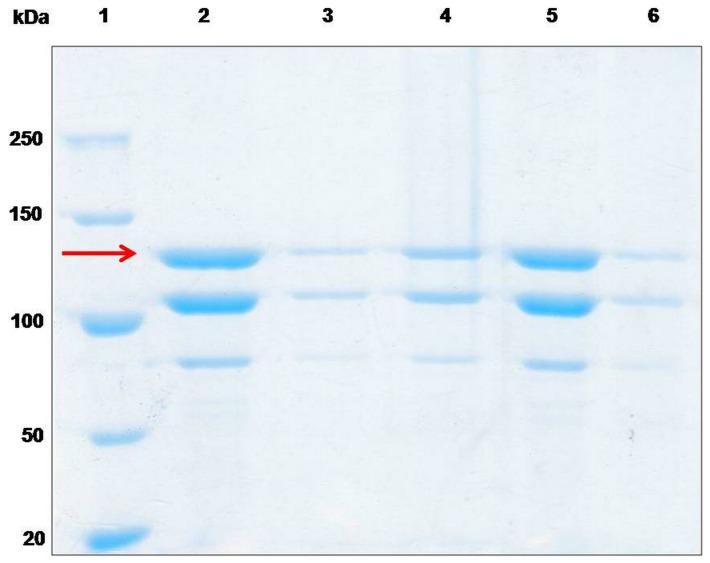
SDS-PAGE of the encapsulation process. Lane 1, protein molecular weight marker. Lane 2, protoxin of Cry1Ac before being mixed with the microcapsules. Lanes 3–4, suspension and deposition together with capsules while loaded at pH 3. Lanes 5–6, suspension and deposition together with the capsule while loaded in water.

The isoelectric point of Cry1Ac protoxin was 4.84. Therefore, the loading process was carried out in the medium where the protoxin was positively or negatively charged at room temperature. The negatively charged inner environment of the microcapsules existed in both of the acidic and alkaline solution as a result of the addition of PSS. In our initial trial, we found that the widely used Bradford method to determine the concentration of the protein could not be used in our system since substances in the solution interfered with the absorbance measurements. Because the structure of microcapsules was destroyed when the sample was heated in boiling water, we used SDS-PAGE to analyze the protein concentration, in order to avoid the absorbance interference. Bovine serum albumin (BSA) was used as a standard to estimate the concentration of the encapsulated protoxins. The result of the loading capability detected by SDS-PAGE (Fig. 2) was coincident with the theoretical electrostatic attraction. The amount of the protoxins indicated by the red arrow in Fig. 2 was different in each sample. Most protoxins accumulated in the microcapsules under acidic conditions remaining in suspension accordingly to the small amount of protoxins and the greater amount of proteins in the deposition, as shown in Fig. 2 (lane 3 and lane 4). Some of the protoxins were lost during the water washes. In contrast, approximately equivalent amounts of proteins were detected in the neutral suspension, and few proteins were in the capsule mixture, as observed within lane 5 and lane 6 (Fig. 2). Thus, the microcapsules presented higher encapsulation efficiency at pH 3. Moreover, we found that the microcapsules loaded with proteins could be dispersed in solution under rapid shaking without the appearance of bubbles, which are produced in normal protein solutions, indicating the effectiveness of the encapsulation.

**Figure 3:**
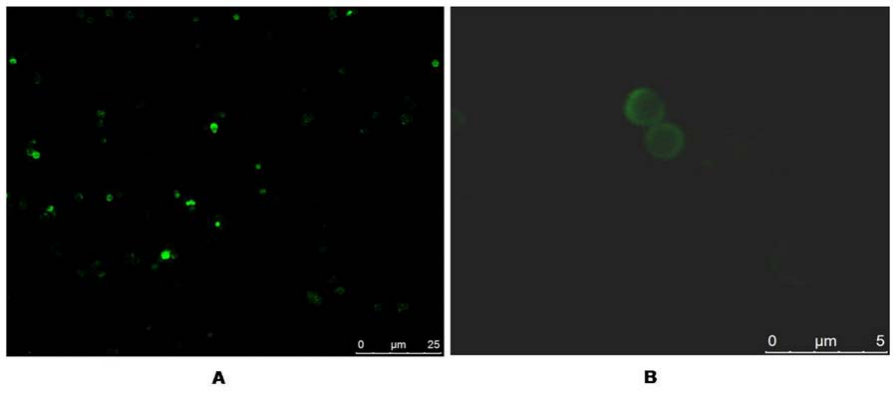
CLSM photos of the FITC-Cry1Ac loaded microcapsules.

To further determine the encapsulation effectiveness, CLSM was used to detect the loading result of the FITC-Cry1Ac microcapsules. Cry1Ac was first labeled by FITC and then encapsulated. The images showed that the microcapsules emitted green ﬂuorescence light with a wavelength of 488 nm and that the fluorescence emission appeared inside the capsules, demonstrating that the FITC-protoxins were successfully encapsulated inside of the microspheres (Fig. 3). A similar size of the microcapsules was confirmed by comparing with the SEM images described above.

**Figure 4:**
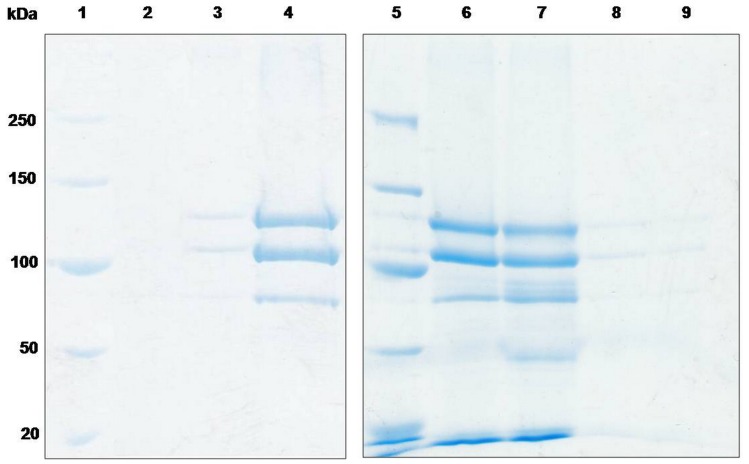
Release experiment detected by SDS-PAGE. Lane 1 and Lane 5, protein molecular weight marker. Lane 2, suspension of capsules loaded at pH 3. Lane 3, suspension of capsules in 50 mM, Na_2_CO_3_, pH 10.2. The protoxins were loaded at pH 7. Lane 4 and Lanes 6–8, releasing suspension of capsules loaded at pH 3. Among them, Lane 4, Lane 6 and Lane 7, releasing suspension in 50 mM Na_2_CO_3_, pH 10.2 for 2 hours, 30 hours, and 40 hours, respectively. Lanes 8–9, releasing suspension in water for 30 hours and 40 hours, respectively.

According to the results above, we chose to load Cry1Ac at pH 3 to obtain more encapsulated protoxins for the subsequent release experiment. The pH was used to trigger the release of the encapsulated Cry1Ac protoxins, and the results were detected by SDS-PAGE as shown in Fig. 4. The sample was washed several times before being delivered into the release buffer to confirm that no protein existed in the suspension. The release was almost invisible at pH 7 after 2 hours, and almost no proteins could be found in the suspension after over 40 hours. Nearly all of the substance inside of the microcapsules were released into the suspension after being transferred into Na_2_CO_3_ buffer (pH 10.2) 2 hours after the loading process. Thus, the release of Cry1Ac protoxins from the PAH/PSS microcapsules made on the CaCO_3_ templates was controlled by pH. In summary, the protoxins can be stably enclosed in the microcapsules in normal neutral conditions and released at pH 10.2, which is the pH of the target insects’ midgut. Among the target insects of Cry1Ac, the Asian corn borer was chosen for use in the insecticidal bioassay. Before the bioassay, all of the samples were stored in water at 4°C for over 10 days, and no protoxins were released during this period. The microcapsules containing Cry1Ac protoxins showed normal insecticidal activity against the Asian corn borer (Table 1). The LC_50_ of the microcapsules containing Cry1Ac against the Asian corn borer was 0.32 µg/g, which is statistically equivalent to the toxicity of unformulated Cry1Ac. As the negative control, hollow capsules without proteins and water shown similar mortality which was 8.3% and 6.3%, respectively.

**Table 1:** Bioassay results of the microencapsulated proteins’ effect on the Asian corn borer.

Samples	LC50 µg/g	Confidence limits (95%) µg/g
**Microcapsules with Cry1Ac**	**0.32**	**0.24–0.42**
**Free Cry1Ac protoxins**	**0.19**	**0.11–0.30**

**Figure 5:**
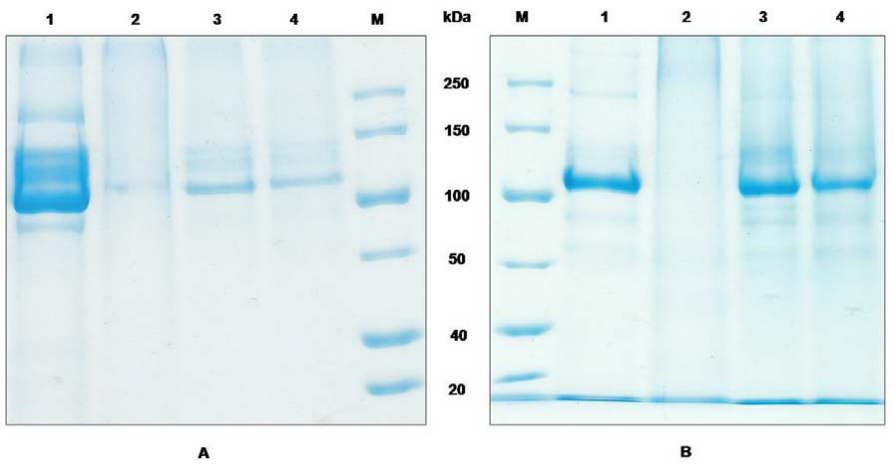
Stability of free and encapsulated Cry1Ac after heat treatment. A: Lane 1, Cry1Ac protoxins stored at 4°C. Lane 2, microcapsules with Cry1Ac stored at 37°C for 5 days. Lane 3, microcapsules with Cry1Ac stored at 4°C. Lane 4, Cry1Ac protoxins stored at 37°C for 5 days. B: Lane 1, Cry1Ac protoxins stored at 4°C. Lane 2, Cry1Ac protoxins stored at 50°C for 5 days. Lane 3, microcapsules with Cry1Ac stored at 4°C. Lane 4, microcapsules with Cry1Ac stored at 50°C after 5 days.

**Figure 6:**
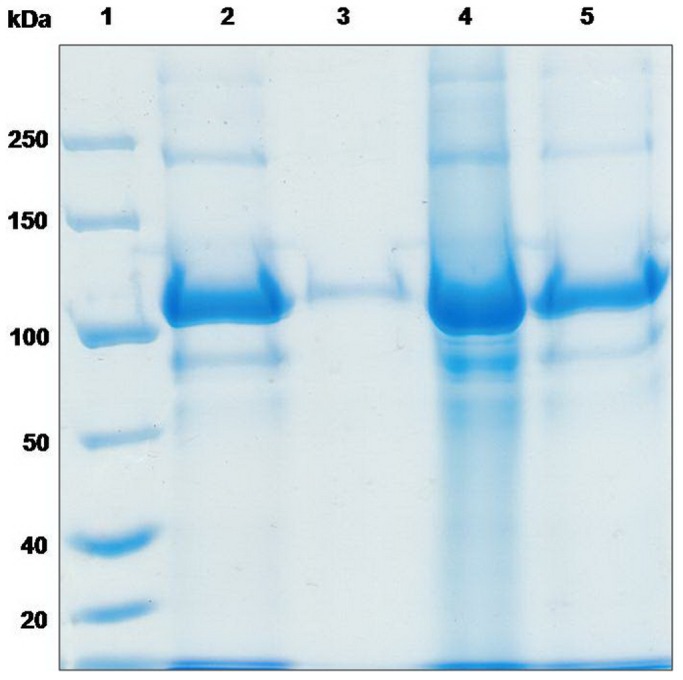
Effect of desiccation on protoxins and microcapsules. Lane 1, protein molecular weight marker. Lane 2, Cry1Ac protoxins. Lane 3, after desiccation at room temperature, the protoxins were redissolved in an equal amount of 50 mM Na_2_CO_3_ buffer. Lane 4, microcapsules with Cry1Ac protoxins. Lane 5, releasing suspension of the microcapsules after desiccation at room temperature and redissolution in 50 mM Na_2_CO_3_ buffer.

The persistence of microcapsules was analyzed to the factors of heat and desiccation. Most of the protoxins degraded after incubation at 37°C for 5 days (Fig. 5a, lane 2) and an thoroughly degradation appeared after the protoxins were kept at 50°C for 5 days (Fig. 5b, lane 2). Compared with the protoxins, there were scarcely changes on the encapsulated protoxins under these temperature conditions (Fig. 5a, lane4; Fig. 5b, lane 4). Bioassay result (Table 2) shows a significant lost of the toxicity of the protoxins after treated for 5 days at 50°C since its mortality with a concentration of LC_50_ is only 3.4% ±4.7, while the encapsulated protoxins still kept a normal mortality rate of 71.1% ±15.7 (Table 2). Desiccation treated results are shown in Fig. 6. It can be seen that most of the protein band of the free protoixn were lost after treated with desiccation, while the encapsulated protoxins still remain its large part. The mortality of the protoxins treated with desiccation decreased to 47.8% ±7.8 (table 2), while the mortality of the encapsulated protoxins fluctuated only in a narrow range before and after treatment. The means between the protoxins and the encapsulated protoxins, either treated with heat or desiccation, were significantly different (P<0.05) by *t*-test (LSD).

**Table 2:** Mortality of microcapsules and protoxins after different treatment on the Asian corn borer.

	% Mortality		
Formulation	Treatment		
	None	Heat*	Desiccation
**Free Cry1Ac protoxins**	**83.3±1.6** a	**3.4±4.7** b	**47.8±7.8** b
**Microcapsules with Cry1Ac**	**65.6±7.8** a	**71.1±15.7** b	**73.4±3.2** b

* Heat: 50°C treated for 5 days.

a The means within a column were not significantly different (P≥0.05) by *t*-test (LSD).

b The means within a column were significantly different (P<0.05) by *t*-test (LSD).

## Discussion

Bio-encapsulated formulation, which can protect pesticides from degradation and provide controllable release of the active ingredient, has been used for decades, and Bt has been encapsulated since the 1960s [22]. A methodology of microencapsulation was investigated which have the capacity to protect the spore crystal aggregate produced by *Bacillus thuringiensis* var. *kurstaki* HD-1 from extreme ultraviolet radiations which are equivalent to 80 and 558 days of sunlight exposure in clear weather in Mexico [23]. These methods have somewhat extended the persistent period; however, the contents of the capsules are released without precise control. Part of the insecticidal Cry toxins can be released gradually from the capsules after the pesticides are sprayed into the environment [11], [23]. Therefore, the efficiency of the protection is not satisfactory. The present study reports a new method to load one Cry protoxin (Cry1Ac) into polyelectrolyte microcapsules and trigger the release by pH changes. In particular, the release process only occurs under alkaline conditions, which seldom exist in the general environment. Electrostatic interaction is the driving force of the spontaneous fabrication and release process.

The shape and size, which should be appropriate for the control of the larval insects, depend on the CaCO_3_ particles. A lot of research in recent years has focused on the crystallization of CaCO_3_ in the presence of additives, including CaCO_3_ morphology and growth rate [24]. Previous studies have demonstrated that simple polyelectrolytes could be used as a tool to control the shape and size of CaCO_3_ particles [25]. Later research [26] reported that the presence of PSS leads to modifications in the structure, morphology and size of the CaCO_3_ crystal, and an increase in the concentration of PSS causes a decrease in the CaCO_3_ particle size. We used PSS addition to successfully construct the CaCO_3_ particles and to build a multilayer of PAH-(PSS-PAH)_2_ on the exterior of the CaCO_3_ (PSS) core through the LbL method. Compared with general templates, such as melamine–formaldehyde (MF), silica and polystyrene particles, CaCO_3_ particles have promising advantages. Their dissolution can be performed under mild conditions by EDTA. In contrast, MF can be dissolved to MF oligomers in the presence of 0.1 M HCl, leading to high mechanical pressure and breakage of the capsule wall [27].

According to the previous study [27], the PSS-doped CaCO_3_ templates have a negative surface because PSS is a negatively charged strong polyelectrolyte. Additionally, the permeation of the hollow capsules depends on the charge of the permeant. Only the positively charged Rd6G could permeate into the interior of the hollow capsules, and similar behavior happens to the few charged chromophores in a labeled dextran [28]. These results show that only the encapsulation of positively charged low molecular weight drugs can be carried out easily in this type of capsule. Thus, the positively charged PAH is the first layer assembled onto the CaCO_3_ particles, and the other four layers are assembled subsequently. After dissociation of the core, the collapsed hollow capsules can disperse into water. As shown in Fig. 2 and Fig. 4, the capsules revealed a pH-sensitive loading and release ability for the Cry1Ac protoxins. Previous experiments showed that capsules composed of PAH and PSS could be switched between an open and a closed state by tuning the pH value. The FITC-labeled dextrans of MW 75000 could penetrate into the capsules at low pH values up to 6 but are not excluded at a pH values higher than 8, owing to the closed state. For other tested proteins, the pH value of the transition between the closed and open state fluctuates with the pH [29]. Our result showing that the protoxins can be encapsulated at pH 3 is consistent with that previous report. However, the release experiment demonstrates an opposite result. We showed that the microcapsules did not remain closed and sustain encapsulation all the time, but the microcapsules released almost all of the proteins when the pH-value increases to 10 (Fig. 4). This different result may be attributed to the different ingredient of the core, which was constructed of MF by Sukhorukov *et al* [29] and CaCO_3_ by us. A negatively charged environment co-determined by CaCO_3_ and PSS is considered as the critical driving force to release the protoxins. Thus, the protoxins were prone to be released at high pH conditions where the protoxins were extremely negatively charged. Furthermore, the result of the SDS-PAGE (Fig. 4) shows that the molecular weight of the proteins did not change after the release process, which is an essential property for the in vivo experiment.

In this study, the resistance of the capsules to some environmental factors was analyzed. It is reported that the longevity of *B. thuringiensis* toxins had a sharply decrease from 5–10°C to 45–50°C [30] and microcapsulation did not provide significant protection against sunlight without UV-protectants. Our result indicated that the protoxins were prone to degrade at high temperature and lose their insecticidal activity, while the microcapsules played a prominent role to protect the encapsulated protoxins. In addition, the encapsulated protoxins shown better stability and toxicity under the condition of desiccation, compared with the free protoxins. Microencapsulation of proteins has been widely studied, but most of the research has stopped at the in vitro process. The maintenance of the biological activity of the proteins is a key point, especially because the proteins are subjected to a broad pH range. The result of the bioassay was encouraging because it showed that the protoxin still kept its toxicity after being loaded into the microcapsules. Although the protection that PSS/PAH shells conferred to α-chymotrypsin from high-molecular-weight inhibitors have been previously studied [31] and urease-loaded PAH/PSS polyelectrolyte capsules have been used to study the fundamental aspects of the biomineralization process [32], this is the first report that shows that PAH/PSS capsules were used to protect proteins through a pH-controlled release method. Importantly, the microcapsules have favorable biological compatibility, and have resistance to certain environmental factors, such as heat and desiccation. Thus this formulation provides a promising methodology to protect protoxins in the environment and to target their toxicity functions only to target insects via the pH of their midguts. Because most Cry protoxins have similar physico-chemical characteristics to Cry1Ac, especially the PI point, this technique can be widely utilized in the future.

## Materials and Methods

### Bacterial Strains and Protoxin Preparation

The Cry1Ac protoxin was expressed in the *B. thuringiensis* HD73 strain. The strain was serially propagated in fresh LB medium before use. *B. thuringiensis* was cultured at 30°C in PB medium (0.5% peptone and 0.3% beef extract) for approximately 40 h. Harvesting of the cells for crystal purification was performed as previously described [33]. The purified protoxin was dissolved in 50 mM Na_2_CO_3_ buffer, pH 10.2.

### Preparation of FITC-Cry1Ac

The Cry1Ac protoxin was labeled by FITC based on a method reported previously [34]. FITC was dissolved in dimethyl sulfoxide, and then the dye solution was added into the protoxin solution (50 mM Na_2_CO_3_, pH 10.2) and stirred at 4°C for one night. The sample was loaded onto a Sephadex G-25 column to remove the free FITC.

### Preparation of Microcapsules

The preparation process was based on a previous study [27] with some modifications. In this experiment, CaCO_3_ microparticles with a monodisperse diameter were prepared as the template. To achieve this, 0.21 g Na_2_CO_3_ and 0.29 g CaCl_2_·5H_2_O were mixed in 20 ml deionized water under magnetic agitation with 0.29 g PSS as an additive. After 30 min, the CaCO_3_ microparticles were collected by centrifugation at 5000 rpm for 10 min and washed in water three times.

The adsorption of polyelectrolytes (2 mg/ml) onto the CaCO_3_ microparticles was performed in 0.1 M Tris-HCl buffer (pH 7.0) for 15 min followed by three washes in water. The PAH layer was deposited by the addition of 15 ml of a 1 mg/ml aqueous PAH solution. The mixture was incubated for 15 min under gentle shaking, and the excess polyelectrolytes were removed by centrifugation at 5000 rpm for 10 min and washed in water three times. After assembly of the four subsequent polyelectrolyte bilayers of PSS/PAH/PSS/PAH, the particles were created by dissolving the CaCO_3_ core in 0.2 M ethylenediaminetetraacetic acid (EDTA) solution (100 mM Tris–HCl buffer at pH 7) for 30 min under agitation and, subsequently, centrifuging the solution at 5000 rpm for 10 min. After washing in water, the hollow microcapsules were re-dispersed in 10 ml Cry1Ac protein solution at different pHs. The mixture was incubated at room temperature for 15 min. The Cry1Ac–loaded microcapsules were obtained after centrifugation at 5000 rpm for 10 min and removal of the suspension.

FITC-Cry1Ac-loaded microcapsules were also obtained follow the same method as above.

### Evaluation of Encapsulated Capability and in vitro Release Experiment

Microcapsules and protein were mixed in either solution at pH 3 or pH 7 (adjusted by acetic acid), and then centrifuge to separate microcapsules by sedimentation. After centrifugation, the supernatant was discarded and samples were resuspended in 10 ml water, and then loaded for SDS-PAGE analysis to estimate the amount of Cry1Ac protoxin remaining into the microcapsules.

The Cry1Ac release from microcapsules experiment was performed by averaging the microcapsules of successful encapsulation into 5 ml water and 50 mM Na_2_CO_3_ buffer, pH 10.2. After incubation for a desired time, the microcapsules were separated by centrifugation, and the protein concentration in the supernatant was determined by SDS-PAGE.

### Characterization

Using an A JSM 6700F (JEOL, JP) microscopy, scanning electron microscopy (SEM) was performed to observe the morphology and to determine the size of the microcapsules with or without the CaCO_3_ core. A drop of each sample was applied onto a sheet of tin foil and dried. Samples were measured by SEM after sputtering with gold. Confocal laser scanning microscopy (CLSM) images were taken with a TCS SP5 (Leica, GER) equipped with a 100× oil immersion objective.

### Resistance to Environmental Factors

#### Heat

The samples of Cry1Ac protoxins and Cry1AC-loaded microcapsules were kept in an incubator at 37°C and 50°C, separately. After 5 days, the samples were detected by SDS-PAGE.

#### Desiccation

The capsules were initially desiccated by filtration and air-dried at room temperature. An equal amount of 50 mM Na_2_CO_3_ buffer was added to redissolve the dried capsules and the solution was centrifuged at 5000 rpm for 10 min to get the suspension. Analogously, the Cry1Ac protoxins were air-dried at room temperature and redissolved in an equeal amount of 50 mM Na_2_CO_3_ buffer. The samples of releasing suspension of microcapsules and redissovled Cry1Ac protoxins were detected by SDS-PAGE.

### Bioassay

#### 50% Lethal Concentration (LC_50_)

Bioassays were performed as described previously [35]. Briefly, insecticidal activities against the Asian corn borer, *Ostrinia furnacalis* (Guenée), were tested by exposing neonate larvae to a diet incorporating bioassay testing seven dilutions of microcapsule-contained Cry1Ac protoxin in water. The diet was uniformly distributed into 48-well trays with a content of 400 mg in each tray. Each well was contained one larva. Cry1Ac protoxin was administered, as a positive control, with the same process. Hollow capsules without proteins and water were used as negative controls. The number of survivors was recorded after 7 days.

#### Mortality of samples of different treatment

According to the data of LC_50_ calculated above, the protoxins and microcapsules of different treatment were diluted to LC_50_ and mixed to the diet of neonate larvae of the Asian corn borer. The diet was uniformly distributed into 48-well trays with a content of 400 mg in each tray. The rest process was same with that above and the mortality of each samples were calculated.
